# Prosthetic-Guided Pterygoid Anchorage for HUG® Subperiosteal Implants: A Technical Report

**DOI:** 10.7759/cureus.114427

**Published:** 2026-08-12

**Authors:** Antoine Diss, Laurine Birault, Augustin Lerebours, Cyrille Grébonval, Antoine Jouclard, Antoine Dubuc, Jérémy Glomet, Christophe Deschaumes

**Affiliations:** 1 Dentistry, Subperiosteal Institute, Nice, FRA; 2 Dentistry, Institut du Sous-Périosté, Nice, FRA; 3 Dentistry, Biotech Dental, Nice, FRA; 4 Biomedical Engineering, Glad Medical, Salon-de-Provence, FRA; 5 Dental Laboratory, Label Dent, Metz, FRA; 6 Odontology, Faculty of Health, Université de Toulouse, Toulouse, FRA; 7 Oral Surgery Unit, Odontology, Centre Hospitalier Universitaire de Toulouse, Toulouse, FRA; 8 Medicine and Surgical Dentistry, Centre Hospitalier Régional Universitaire de Tours, Tours, FRA; 9 Oral Implantology, University of Clermont Auvergne, Clermont-Ferrand, FRA

**Keywords:** digital implantology, graftless rehabilitation, guided surgery, maxillary atrophy, pterygoid implant, subperiosteal implant

## Abstract

Severe maxillary atrophy presents significant challenges for implant-supported rehabilitation due to sinus pneumatization, extensive bone resorption, and reduced posterior anatomical support. Modern digitally designed subperiosteal implants have re-emerged as graftless alternatives enabled by advanced computer-aided design-computed-aided manufacturing (CAD-CAM) workflows and optimized anatomical anchorages. Pterygoid fixation provides valuable posterior stability; however, free-hand drilling carries risks of injury to critical vasculonervous structures, including the descending palatine artery and the pterygopalatine fossa contents.

This technical report describes a novel prosthetic-guided approach to ensure drilling in the pterygoid process utilizing an integrated semi-cylindrical guide incorporated directly within the definitive prosthetic framework of the HUG® (Biotech Dental®, Salon-de-Provence, France) subperiosteal implant.

Postoperative computed tomography (CT) scans superimposed onto preoperative planning data show minimal deviation in apex positioning, as well as minimal angular deviation of the pterygoid screws between planned and executed positions. This technique aims to secure reproducible and accurate pterygoid anchorage while minimizing iatrogenic risk associated with freehand posterior maxillary access in the management of severe maxillary atrophy.

## Introduction

Rehabilitation of the severely atrophic maxilla remains clinically demanding. Advanced resorption of the alveolar process, combined with maxillary sinus pneumatization, often limits the feasibility of conventional endosseous implants without adjunctive grafting procedures such as sinus lift, block grafts, or staged reconstruction. These surgical techniques are associated with increased morbidity, cost, and treatment time and may be poorly accepted or contraindicated in elderly or medically fragile patients. 

To address these limitations, digitally designed, additively manufactured custom subperiosteal implants have been reintroduced as a graftless alternative for full-arch rehabilitation in severely resorbed jaws [1]. Three-dimensional imaging, computer-aided design (CAD), and precision computer-aided manufacturing (CAM) enable intimate adaptation of the titanium framework to basal bone and controlled distribution of functional loads. Recent studies reporting short- to medium-term survival and complication rates demonstrate the appeal, reliability, and acceptable morbidity of subperiosteal titanium implants with CAD-CAM [2-4].

In the context of such modern subperiosteal frameworks, the pterygomaxillary region has gained increasing attention as a strategic anchorage site [5]. Pterygoid fixation enables the engagement of dense cortical bone in the pterygoid process and the posterior maxilla, providing posterior cross-arch stabilization while bypassing critically resorbed alveolar bone [6]. This concept is particularly valuable in patients with Cawood-Howell class V-VI atrophy [7], in whom residual alveolar height is insufficient for conventional implants but basal bone support remains available in the pterygomaxillary complex.

However, the pterygomaxillary region is anatomically complex. The pterygoid plates are in close relationship with the descending palatine artery, the pterygoid venous plexus, and the neurovascular structures of the pterygopalatine fossa neurovascular bundle. Freehand pterygoid drilling entails a non-negligible risk of vascular injury, sinus wall perforation, or misdirection into critical anatomical areas [8].

Recent developments in static and dynamic guided surgery have improved the accuracy of posterior implant placement, including pterygoid implants, by transferring preoperative virtual planning to the operative field [9,10]. These technologies may reduce the angular and linear deviations between planned and actual implant positions and thus mitigate the risk of complications.

To our knowledge, to date, however, no published report has described a prosthetic-integrated guiding structure incorporated directly into a definitive subperiosteal implant-supported full-arch bridge to control pterygoid drilling.

The present technical report describes such an approach using a specific subperiosteal implant protocol (HUG® implant; Biotech Dental®, Salon-de-Provence, France). This custom-made CAD/CAM-manufactured titanium framework is designed for the rehabilitation of patients with severe maxillary or mandibular atrophy.

## Technical report

**Figure 1:**
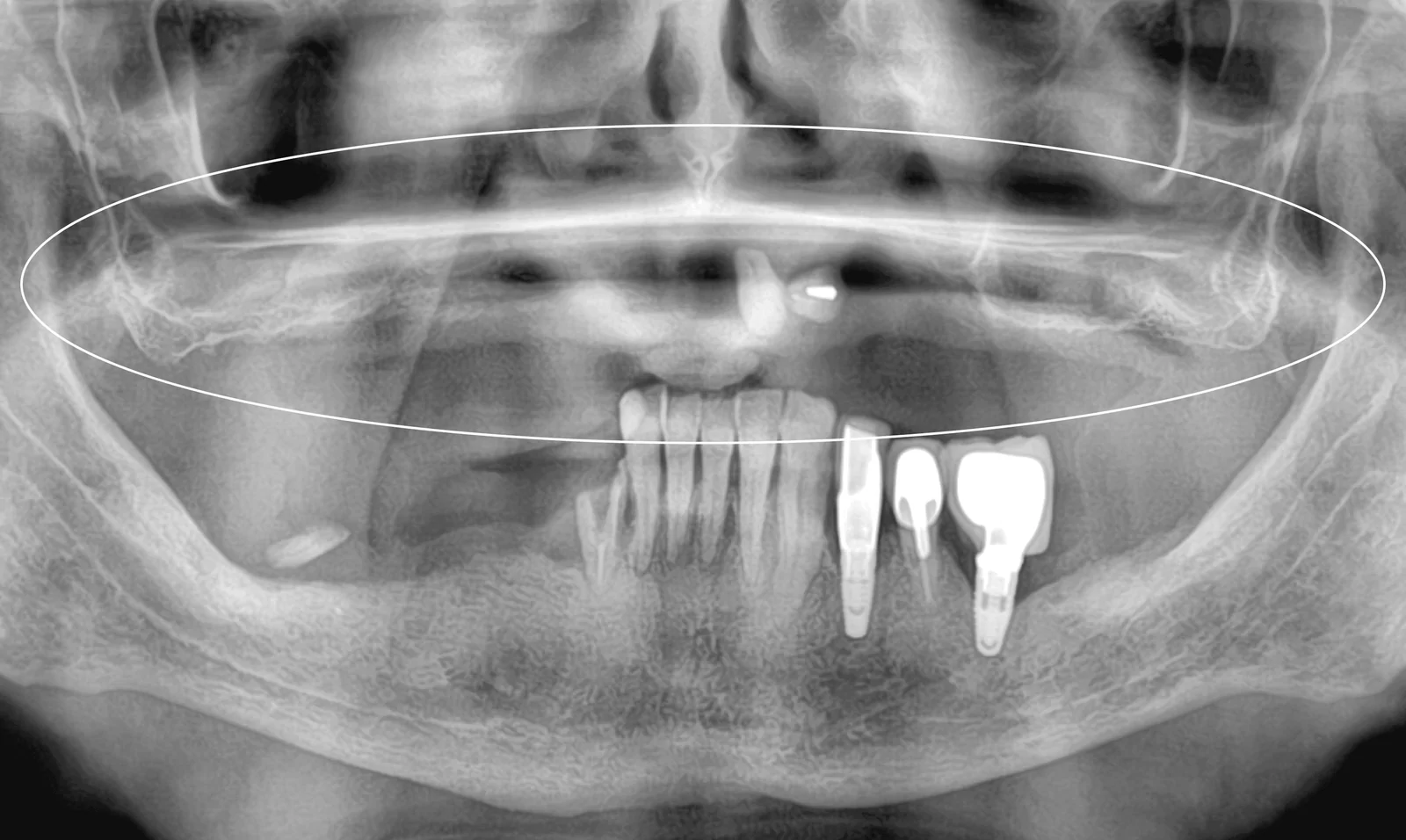
Preoperative panoramic X-ray. The white oval delineates the extent of the residual maxillary bone loss and the area of extreme maxillary atrophy (Cawood–Howell Class VI).

**Figure 2:**
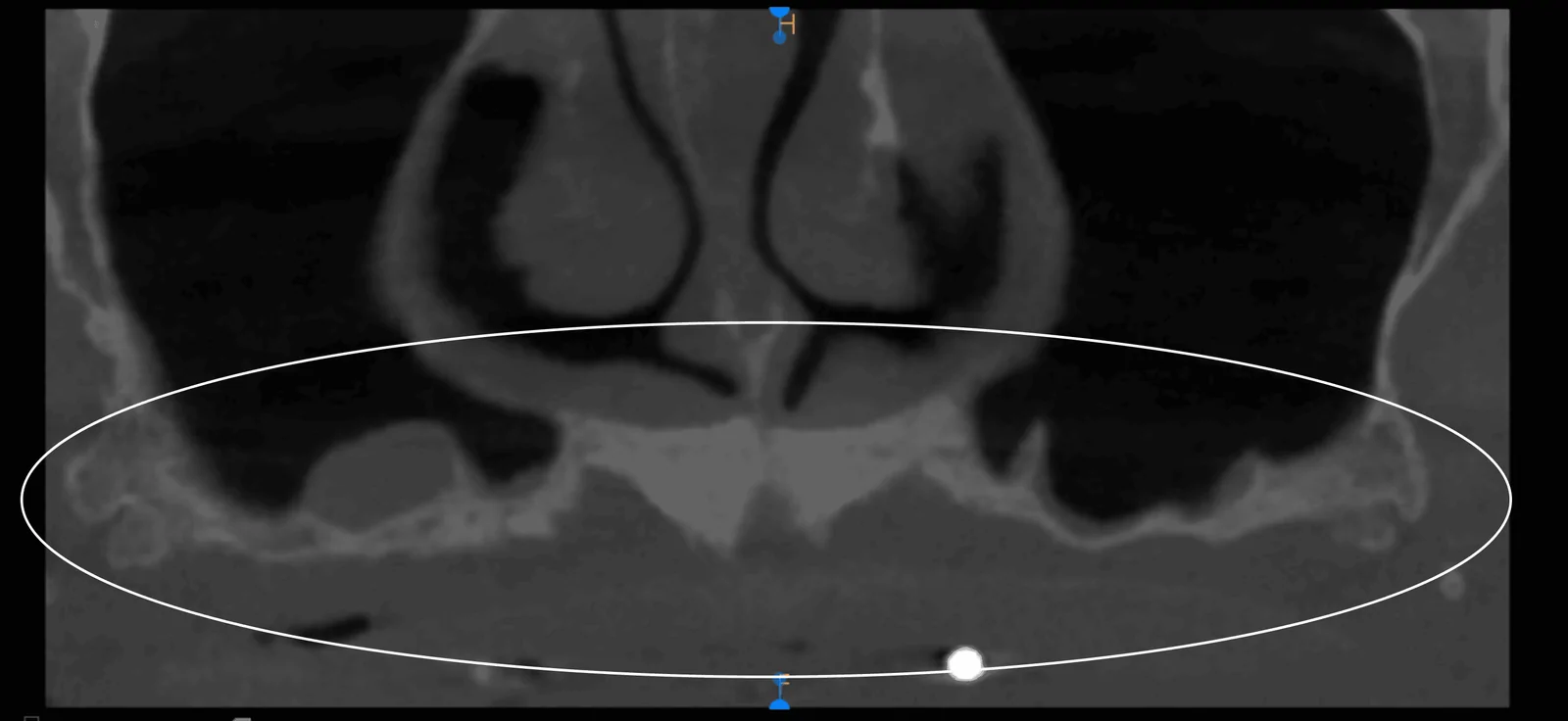
Preoperative CBCT coronal view illustrating advanced maxillary bone atrophy. The outlined region corresponds to the area of severe alveolar ridge resorption.

**Figure 3:**
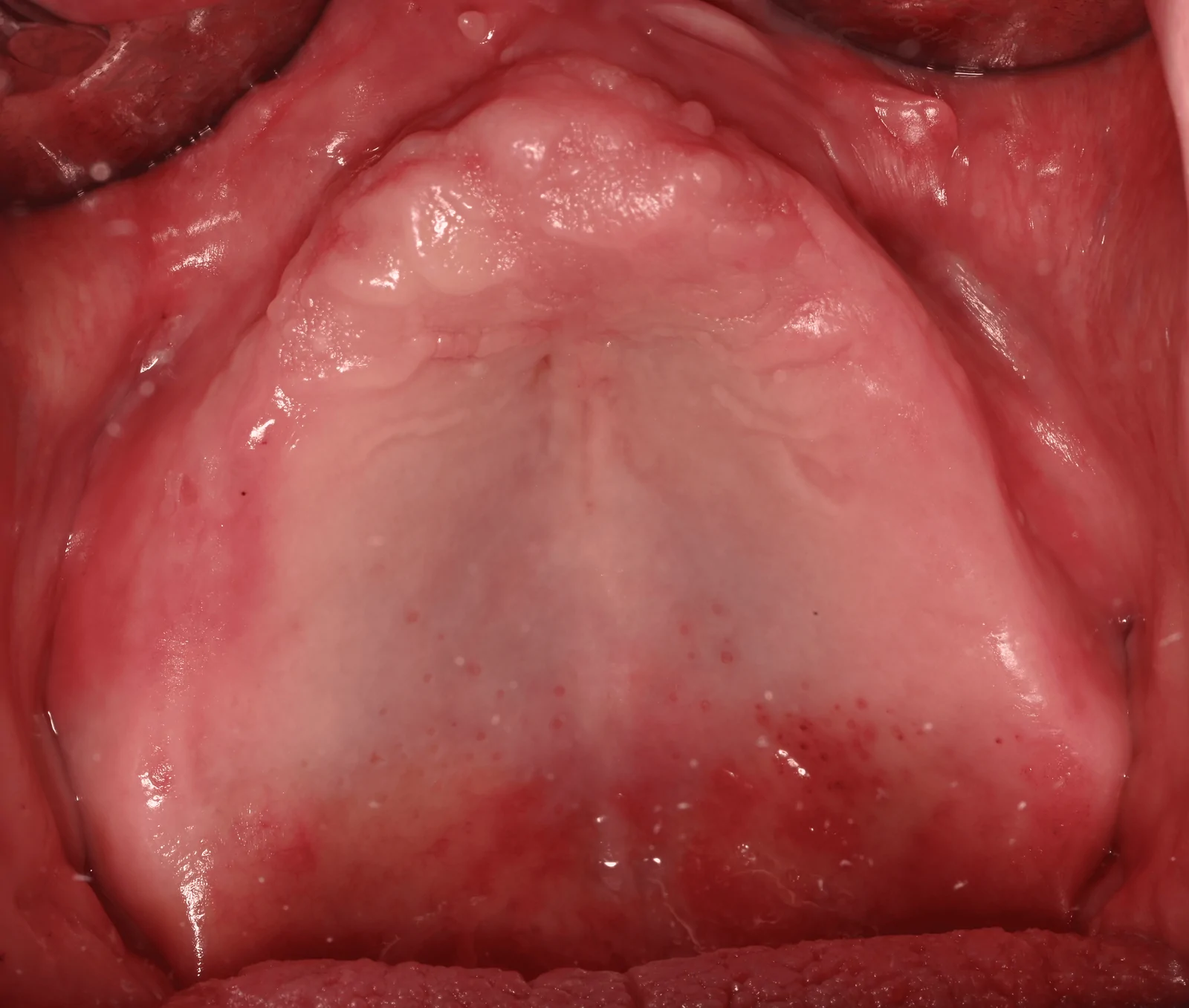
Occlusal view of the fully edentulous maxilla.

An 82-year-old male presented with complete maxillary edentulism and poor tolerance of removable prostheses, reporting impaired mastication and reduced quality of life. The patient reported no relevant medical history, presented with no current systemic pathology, and was not undergoing any medical treatment at the time of intervention. Clinical examination revealed generalized ridge atrophy, and cone-beam computed tomography (CBCT) confirmed Cawood-Howell class VI maxillary resorption, with minimal residual alveolar height in the posterior maxilla and marked bilateral sinus pneumatization (Figures 1-3). The patient declined sinus grafting and expressed a preference for a single-stage, immediately loaded fixed solution. This case was treated, managed, and followed up entirely in a private dental clinic (Subperiosteal Institute, Nice, France). The conceptual development of the prosthetic-guided pterygoid anchorage technique described in this report was not the product of a single institution but emerged from a collective and iterative process of clinical reflection conducted collaboratively among the co-authors. Each contributor brought specific expertise in digital planning, biomechanical engineering, prosthetic design, surgical anatomy, or clinical implantology, which collectively shaped the rationale and design of this approach. All clinical procedures were performed in accordance with the ethical standards of the Declaration of Helsinki.

Digital planning and prosthetic-integrated guide design

A dual-scan protocol [11] was used to acquire anatomical and prosthetic data. Patient-specific maxillary models were generated from CBCT data (CS 9600, Carestream Dental, Atlanta, GA, USA) using 3D Slicer (version 5.8; open-source software for medical image analysis, Surgical Planning Laboratory, Brigham and Women’s Hospital, Harvard Medical School, Boston, MA, USA) [12]. The segmentation was reviewed and manually refined when required to ensure anatomical accuracy, then exported as surface meshes for subsequent design steps.

The existing maxillary prosthesis was scanned separately and in occlusion, and the datasets were merged with the CBCT images. Complementary datasets, including the intraoral scan (NewTom VisioScan WL, Cefla S.C., Imola, Italy) and prosthetic wax-up, were registered to the bone coordinate system using radiopaque markers incorporated in the dental guide, thereby ensuring accurate prosthetically driven planning. All registered models were imported into Blender® (version 4.8; Blender Foundation, Amsterdam, Netherlands) for implant planning [13].

Two patient-specific HUG® titanium subperiosteal implants were designed to rest on basal bone pillars, including the canine and zygomatic buttresses and the pterygomaxillary sutures, with posts emerging in prosthetically driven positions. Both implants were fixated using six osteosynthetic screws: three in the anterior maxilla, two in the zygomatic region, and one in the pterygoid process. Screw trajectories were planned to avoid critical anatomical structures, particularly the maxillary sinus, while achieving bicortical fixation and ensuring accessibility under limited mouth opening. Final positioning of abutments and screws was validated by the surgeon.

Implant modeling was performed in HyperMesh 2025® (Altair Engineering Inc., Troy, MI, USA) using the segmented bone surface meshes as the basis for the geometric reconstruction [14]. A triply periodic minimal surface (TPMS)-Gyroid lattice structure [15] with a unit cell size of 2 mm was incorporated within the main branch to promote bone ingrowth while maintaining structural integrity. After final validation by the oral surgeon, the implants were printed (ProX DMP 200, 3D Systems, Rock Hill, SC, USA) using direct metal laser sintering (DMLS) with medical-grade titanium alloy (Ti-6Al-4V ELI, Grade 23). The abutments were then machined (G5 milling machine, Dental Machine S.r.l., Bobbio, PC, Italy). The implant was decontaminated (LLLE6 machine, FISA, Milan, Italy) and underwent anodization to impart color without impairing osseointegration. The use of color is particularly valuable in the case of gingival retraction for esthetic reasons. Finally, the implant was delivered clean but non-sterile and was sterilized under the responsibility of the oral surgeon using a validated Class B steam autoclave with a prion decontamination cycle (steam sterilization at 134°C for 18 minutes, followed by the manufacturer's recommended drying phase), ensuring full sterilization traceability.

**Figure 4:**
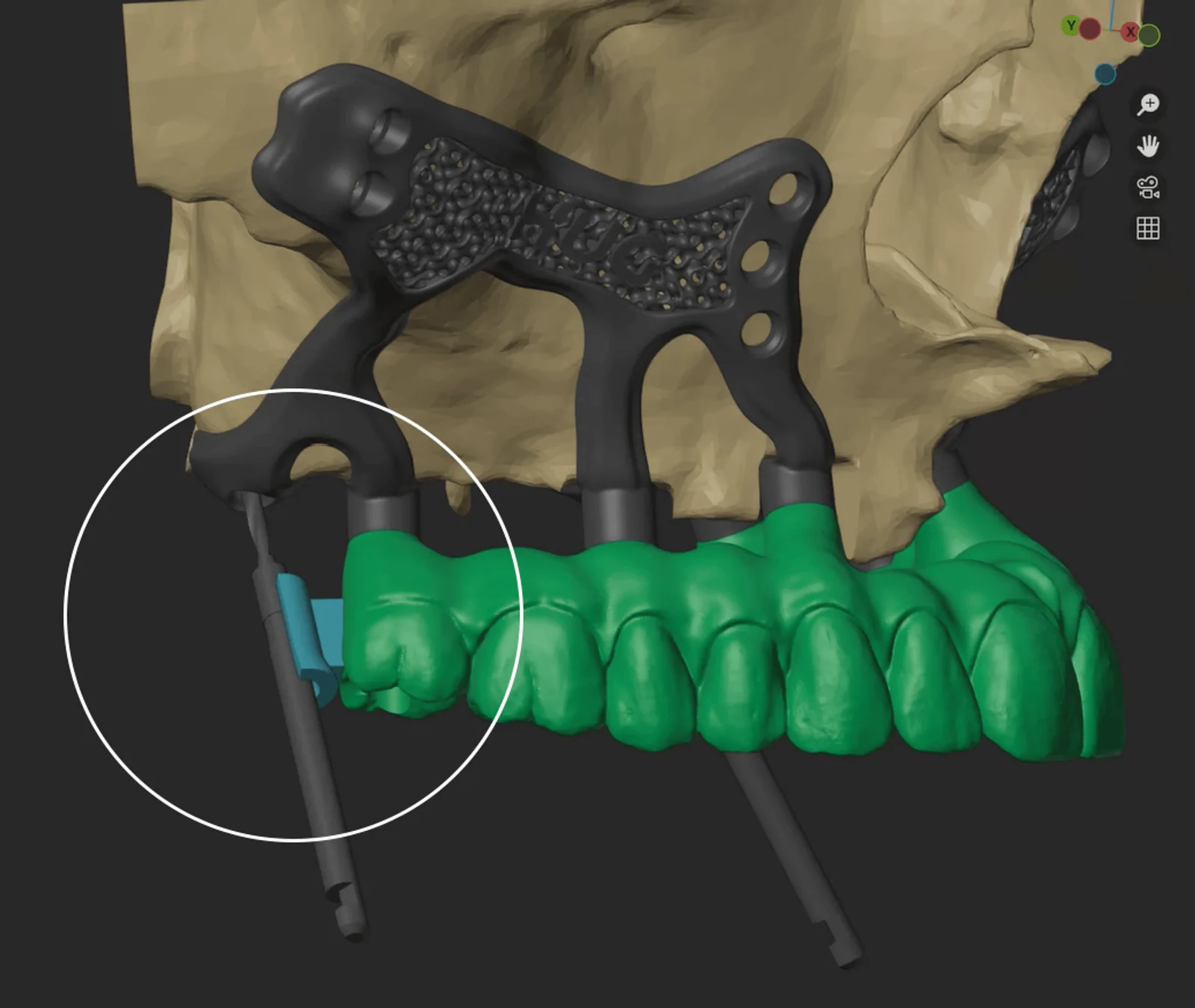
Semi-cylindrical distal channel integrated into the prosthetic design to guide the pterygoid drilling trajectory. The circled area highlights the channel used for drill alignment during pterygoid fixation. The figure was generated using Blender® (Blender Foundation, Amsterdam, Netherlands).

**Figure 5:**
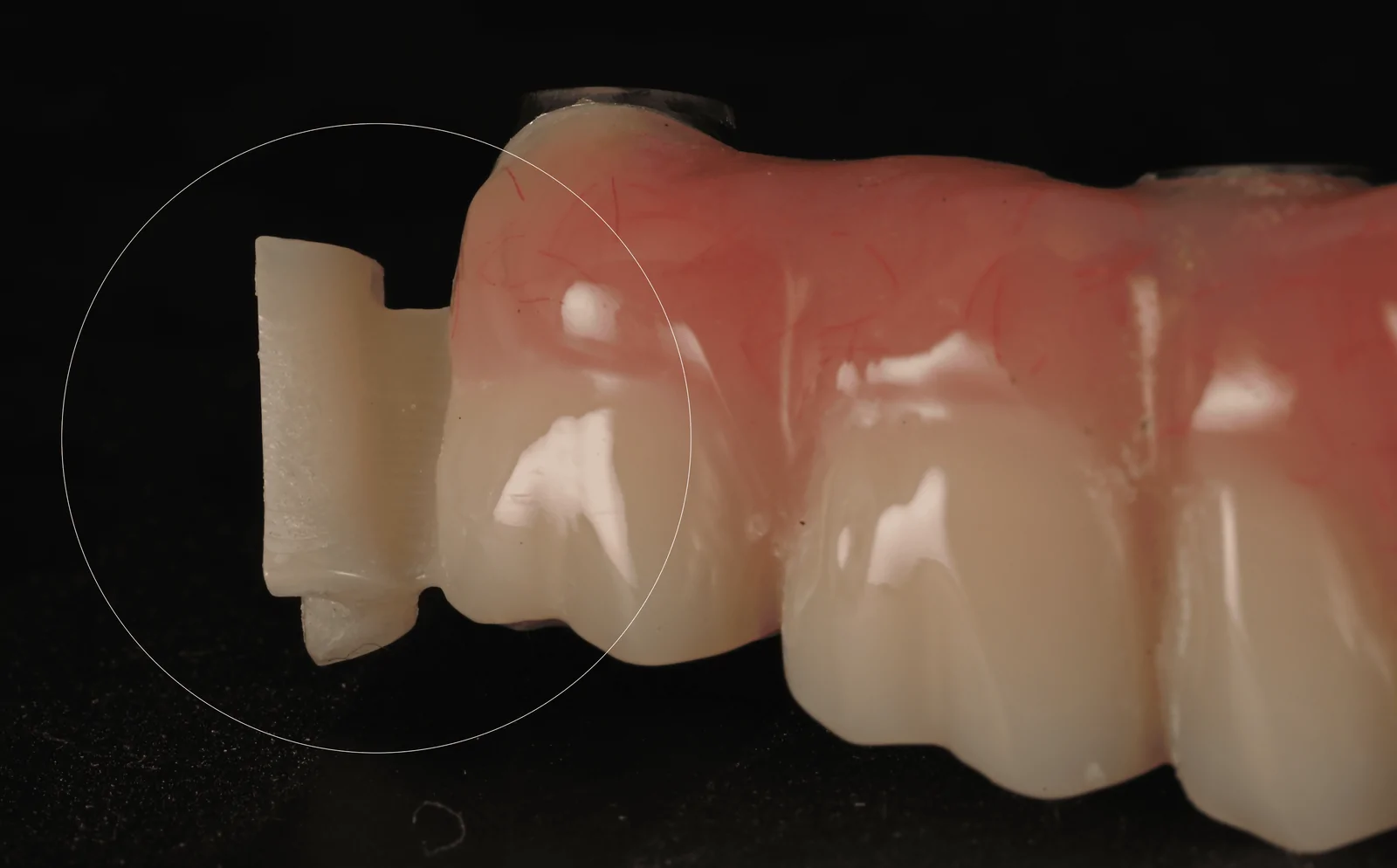
Close-up view of the distal semi-cylindrical channel integrated into the prosthetic design. The circled area highlights the drill-guiding feature used to control the pterygoid drilling trajectory.

**Figure 6:**
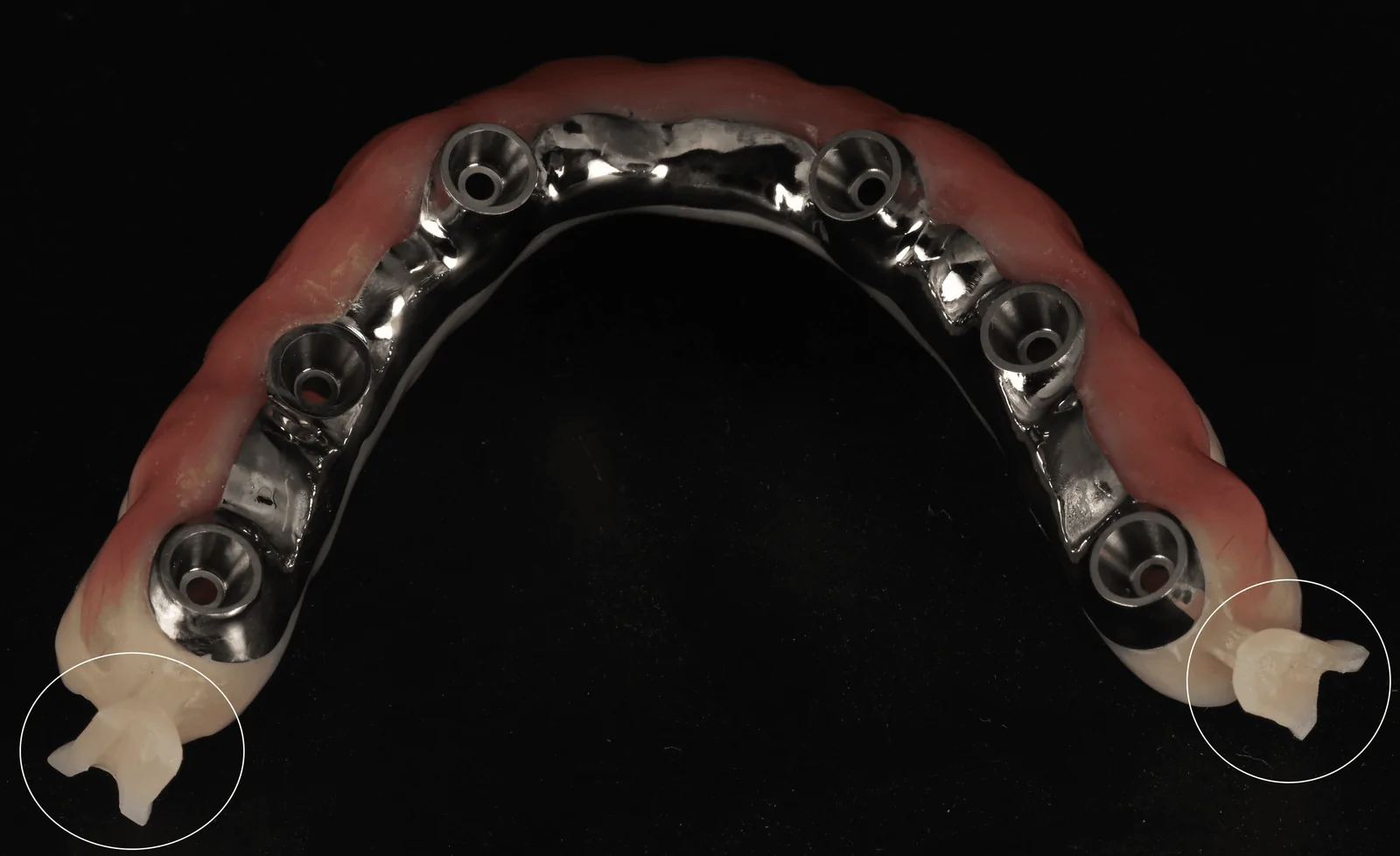
Posterosuperior view of the full-arch prosthesis showing the titanium framework, printed PMMA dentition, and two semi-cylindrical guiding sleeves for pterygoid drilling. The circled areas highlight the drill-guiding features located in the distal prosthetic extensions. PMMA: polymethyl methacrylate

**Figure 7:**
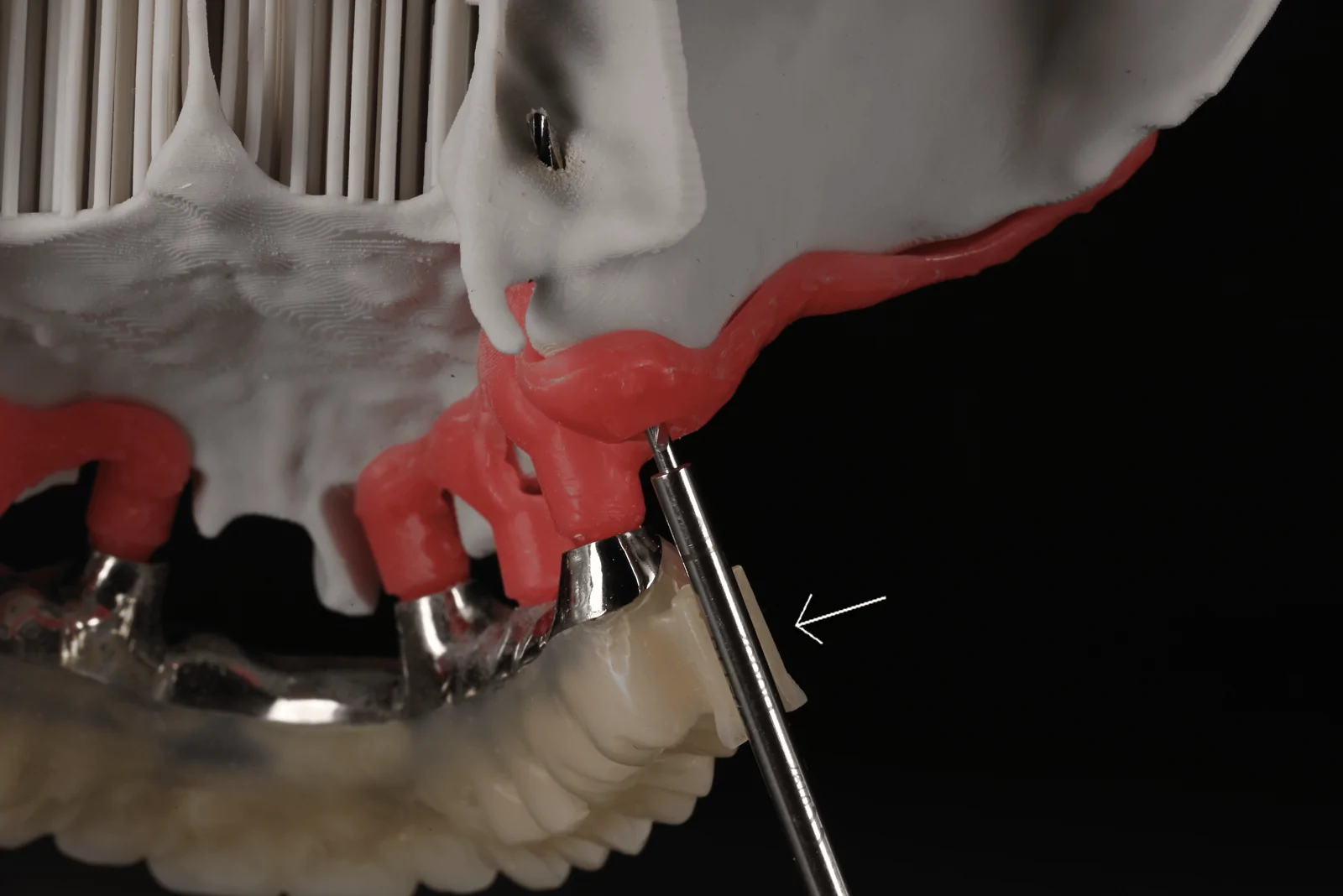
Maxillary model with the full-arch prosthesis positioned on PMMA-printed implant replicas. The integrated drilling guide directs the pterygoid osteotomy, with the drill tip emerging at the center of the pterygoid process (arrow). PMMA: polymethyl methacrylate

**Figure 8:**
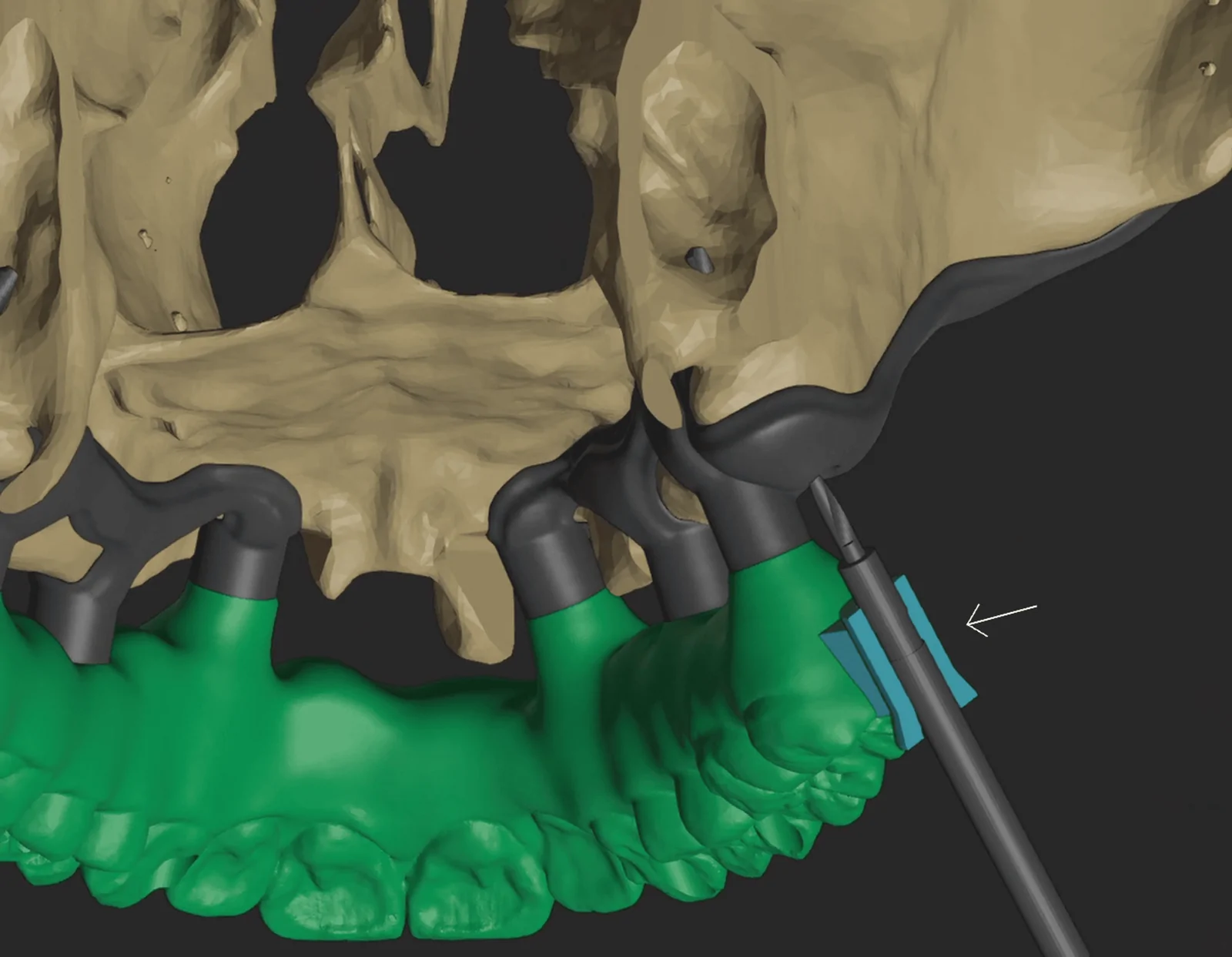
Posterior view of the pterygoid drilling simulation. The arrow indicates the concordance between the virtually planned trajectory and the drill position obtained using the integrated surgical guide on PMMA-printed implant replicas. The figure was generated using Blender® (Blender Foundation, Amsterdam, Netherlands). PMMA: polymethyl methacrylate

The definitive full-arch prosthesis was virtually designed on the subperiosteal framework. In the distal posterior segments, a semi-cylindrical “half-tube” channel was integrated into the prosthetic design and aligned with the planned pterygoid screw trajectory (Blender 4.3®) [13]. This channel allowed guided passage of a 1.5-mm pilot drill (Adapsia, Lyon, France) to achieve cortical engagement in the pterygoid process, facilitating safe and reproducible anchorage (Figure 4). The definitive prosthesis and integrated guide were manufactured as a single polymethyl methacrylate (PMMA) structure bonded to a titanium framework (Figures 5-8).

Surgical procedure

Surgery was performed under local anesthesia. A palatally offset crestal incision was performed to preserve keratinized gingiva on the buccal aspect of the future implant connection. A full-thickness mucoperiosteal flap was raised to expose the anterior nasal spine region, canine pillars, zygomatic buttresses, and posterior maxillary tuberosities.

**Figure 9:**
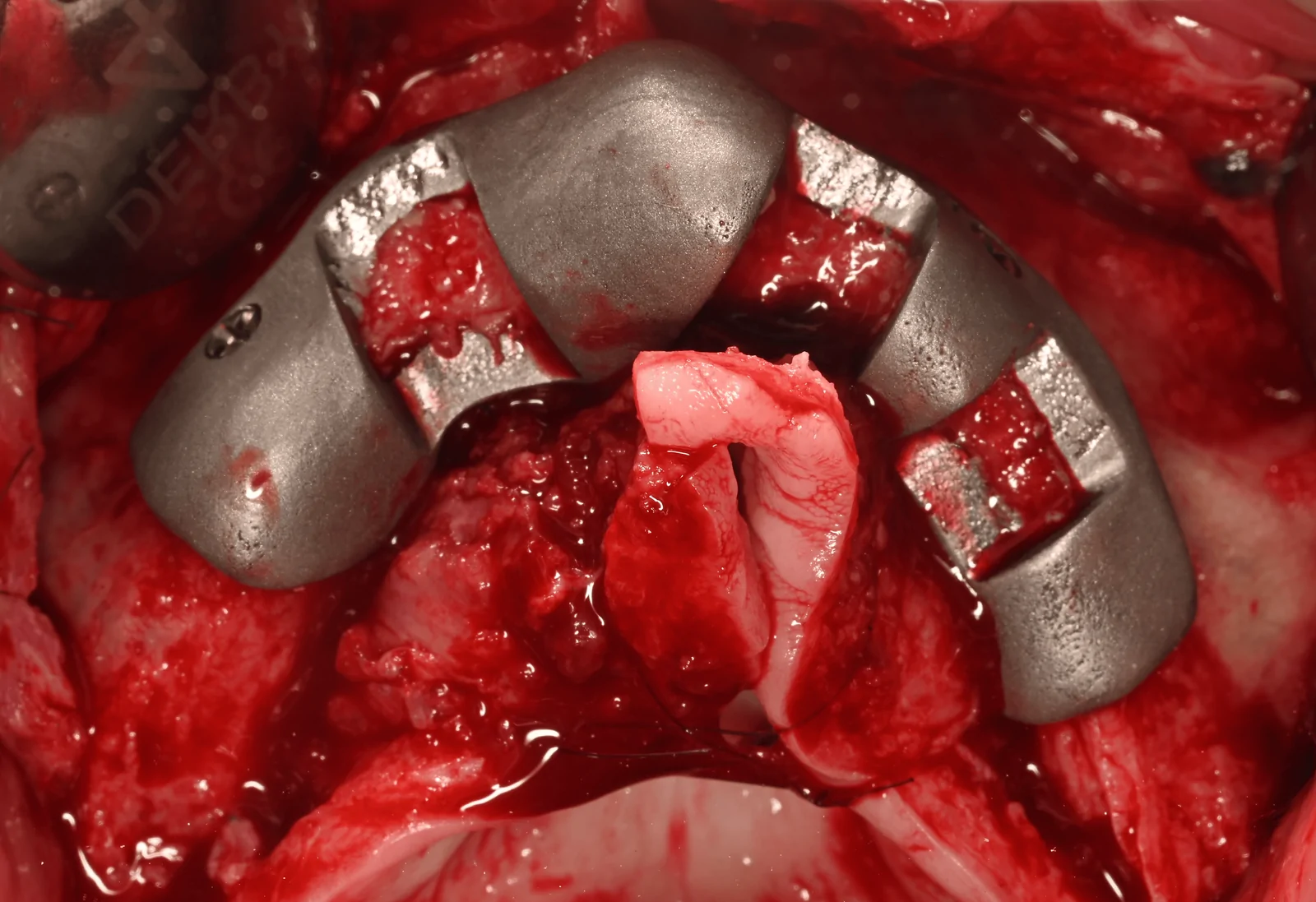
Bone-cutting guide positioned to regularize premaxillary ridge irregularities and create a receptacle for pillar seating, promoting integration and minimizing soft-tissue recession.

A bone-cutting guide was positioned intraoperatively to regularize localized irregularities of the maxillary ridge and create a controlled osteotomy for the premaxillary support pillars (Figure 9). This guided bone reshaping enabled the pillars to seat within a predefined osteotomy rather than on an irregular or convex bony surface, thereby improving the accuracy and stability of their positioning.

**Figure 10:**
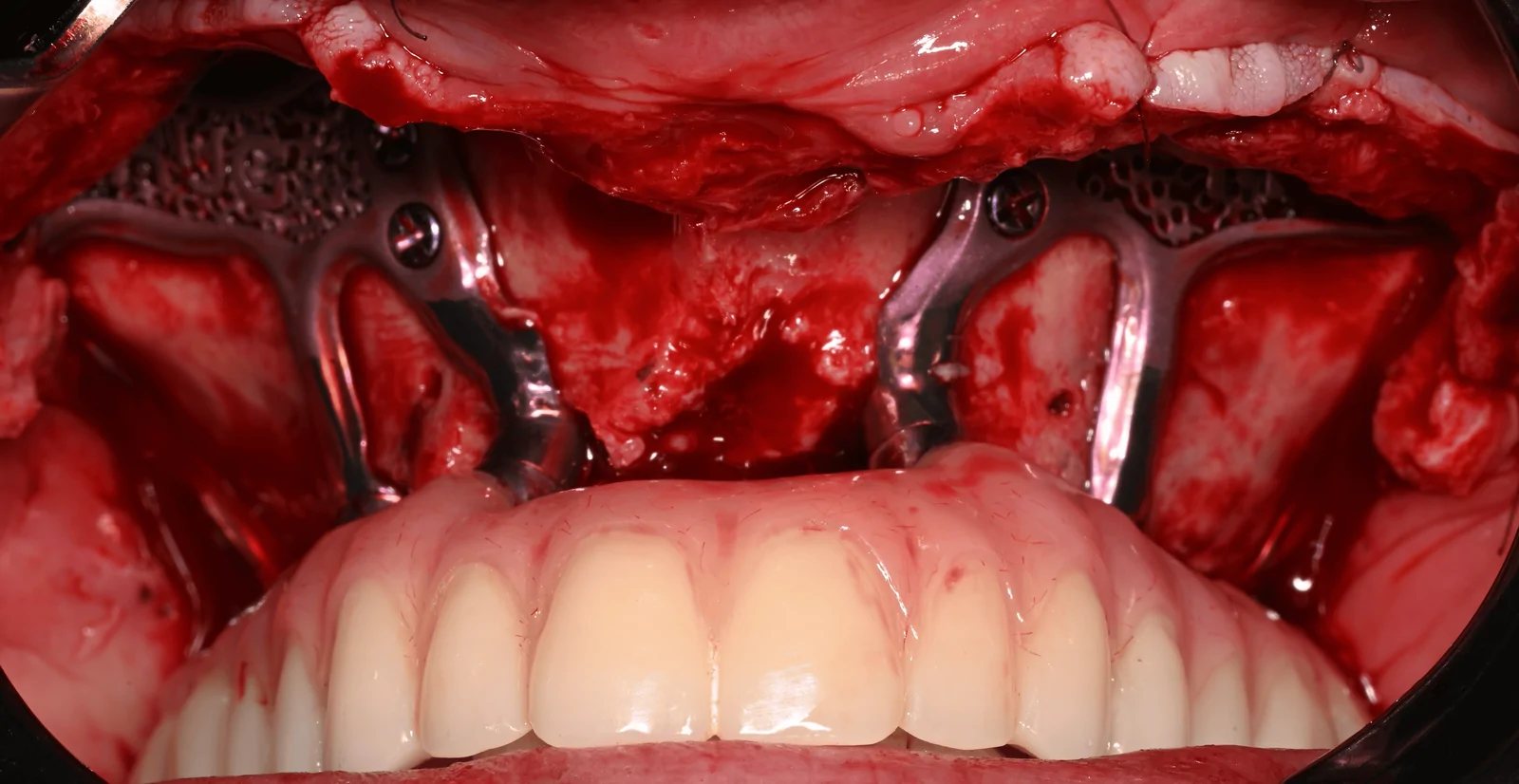
Two HUG® subperiosteal implants fixed to the maxilla, connected by the definitive screw-retained bridge. HUG® (Biotech Dental®, Salon-de-Provence, France)

The two subperiosteal implants, consisting of titanium frameworks, were positioned and verified for passive adaptation. The definitive bridge was then secured to each pillar with screws, and the patient was instructed to bite to ensure proper occlusal positioning. Initial fixation was achieved with screws at the canine and zygomatic buttresses, providing primary stabilization of the subperiosteal implant and screw-retained fixed prosthesis (Figure 10).

Once the framework and bridge were secured anteriorly and laterally, the prosthetic-guided pterygoid drilling was performed. A 1.5 mm drill was introduced through the semi-cylindrical guide channel on the distal aspect of the bridge, and the osteotomy was advanced towards the planned pterygoid target region under irrigation. Preoperative radiographic planning established a target osteotomy depth of 14 mm. During surgery, the drills used did not incorporate depth markings; therefore, osteotomy depth was assessed and controlled through tactile feedback and intraoperative evaluation before placement of the 14-mm fixation screw. The same procedure was repeated contralaterally.

After completion of the osteotomies, the half-tube guide segments were removed mechanically with a rotary instrument, without disturbing the overall position of the framework. Two fixation screws (2.0 mm in diameter, 14 mm in length according to the preoperative radiographic planning) were then inserted bilaterally into the pterygoid region through the prepared trajectories. Insertion torque was approximately 25 N.cm.

**Figure 11:**
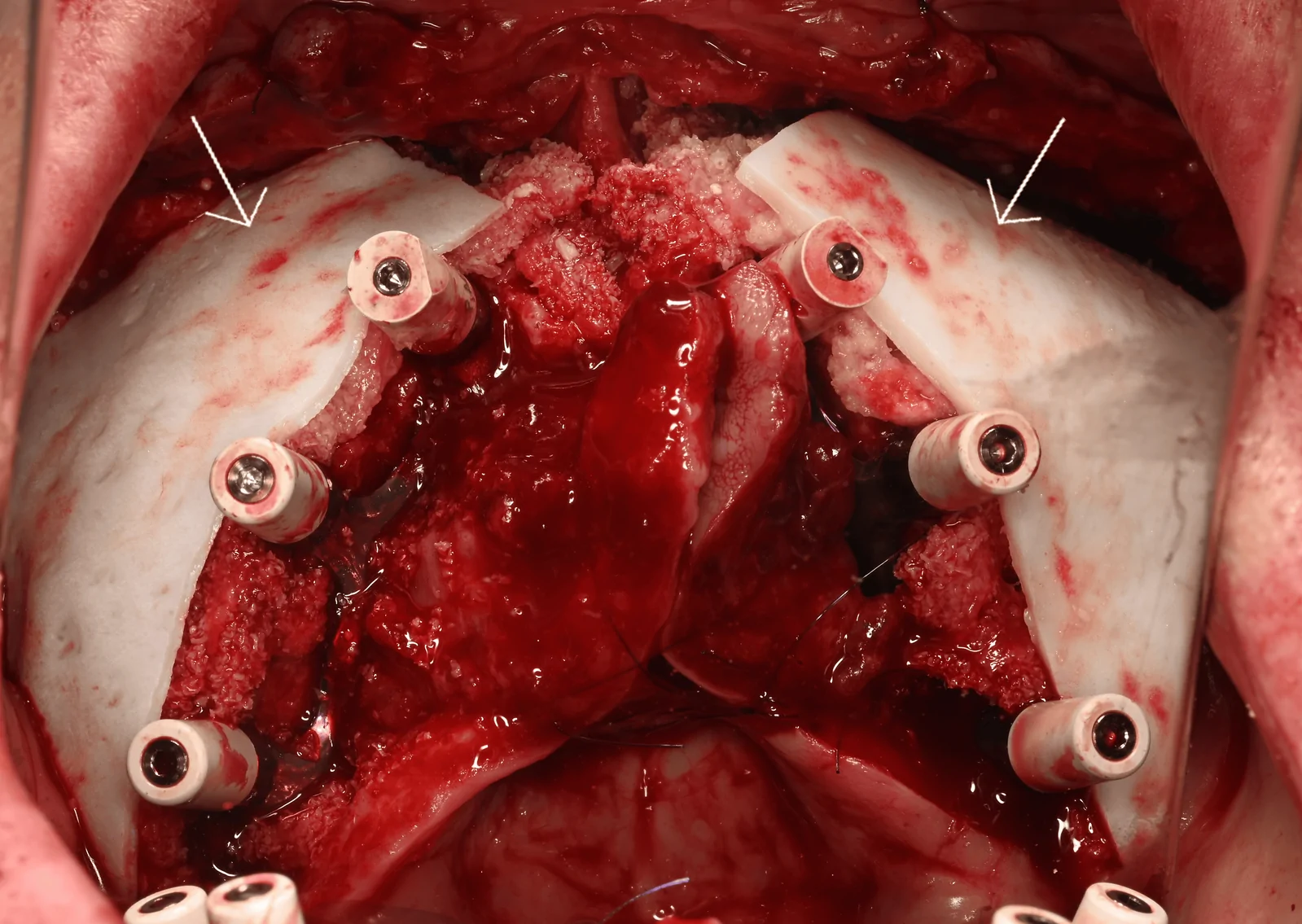
Vestibular grafting around the subperiosteal implant pillars using particulate bone substitute covered with acellular porcine-derived dermal matrices (arrows).

After placement of all fixation screws (canine, zygomatic, and pterygoid), the bridge was temporarily removed to allow placement of a xenogenic particulate bone substitute (The Graft®, Purgo Biologics, Seongnam-si, South Korea) in selected areas beneath the framework, followed by coverage with an acellular dermal matrix derived from porcine tissue (Novomatrix®, BioHorizons, Birmingham, AL, USA) to thicken the soft tissue. The membrane was stabilized without the use of fixation pins or screws, relying on passive adaptation and stabilization by the surrounding tissues. After periosteal releasing incisions were performed with a No. 15 scalpel blade, tension-free closure was obtained through controlled flap advancement. Closure was achieved using resorbable 5-0 monofilament sutures, including deep U-shaped mattress sutures circumferentially placed around each prosthetic pillar to secure the flap and superficial O-shaped interrupted sutures between adjacent pillars to optimize soft tissue adaptation and sealing (Figure 11).

**Figure 12:**
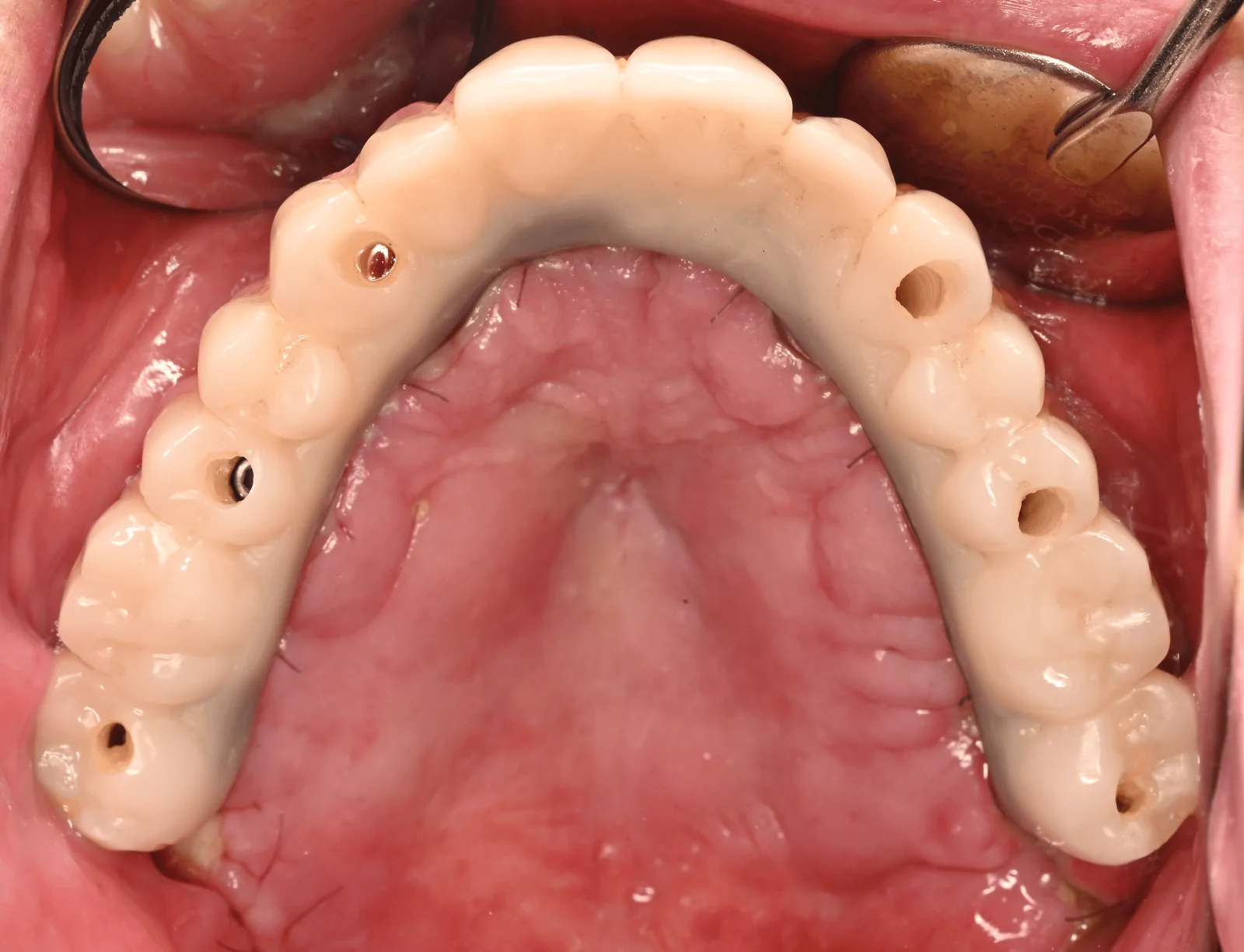
Occlusal view (at seven days after surgery) of the screw-retained fixed prosthetic bridge secured to the subperiosteal implants.

The prosthesis was then reinserted and tightened (Figure 12). Two control radiographs were obtained: one panoramic radiograph and one CBCT exam.

Postoperative evaluation

**Figure 13:**
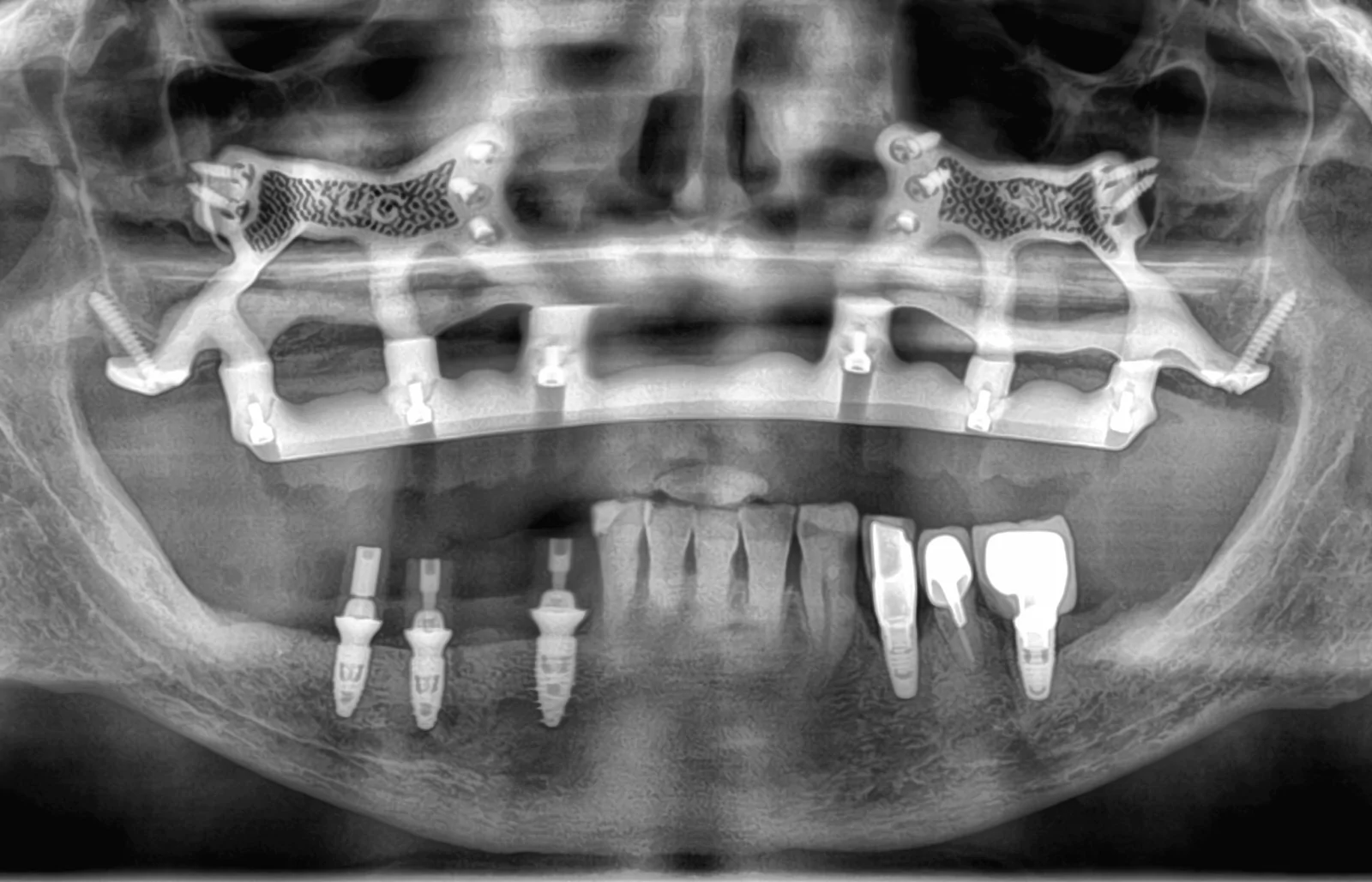
Postoperative panoramic X-ray imaging.

At the two-week postoperative follow-up, healing was uneventful, with no evidence of hemorrhagic events, neurological complications, or infection (Figure 13).

To evaluate the accuracy of screw placement relative to the planned angulation, the planned screw models were registered to the post-implantation CBCT coordinate system. To do so, anatomical structures that remained unchanged between pre- and post-implantation imaging, including the maxillary sinuses and the posterior maxillary region, were segmented from both datasets using 3D Slicer (version 5.8; open-source software for medical image analysis, USA) [12]. The resulting segmentations were exported as surface meshes.

Then, the rigid transformation aligning the pre-implantation imaging (used for implant and screw planning) to the post-implantation imaging was computed using an Iterative Closest Point (ICP) algorithm [16] applied to the corresponding anatomical surface meshes. This transformation matrix was then applied to the planned pterygoid screw models, thereby registering them into the post-implantation CBCT coordinate system.

**Figure 14:**
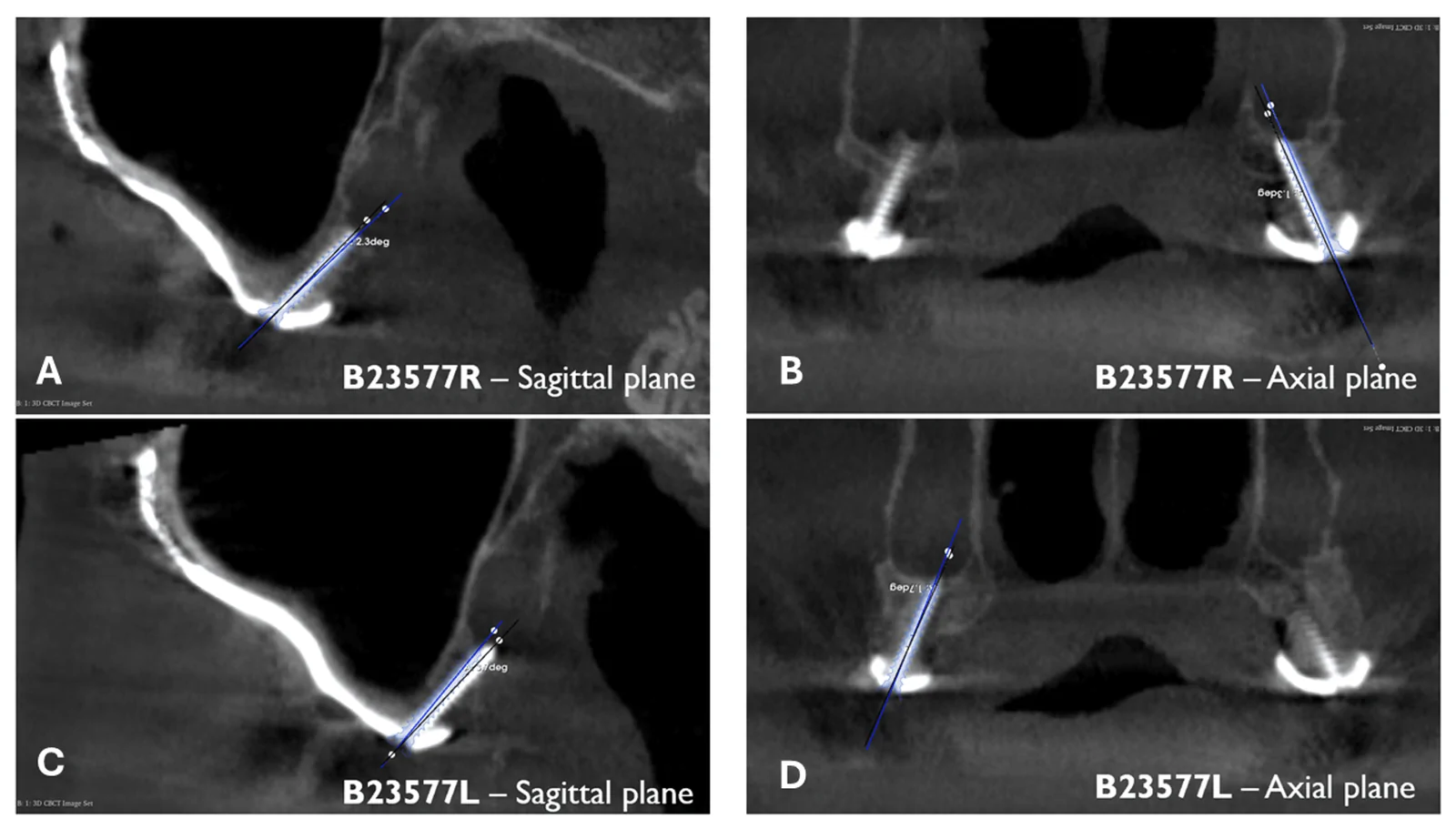
Comparison between virtual planning and postoperative pterygoid screw positions. A: sagittal CBCT view of the right side (B23577R); B: axial CBCT view of the right side (B23577R); C: sagittal CBCT view of the left side (B23577L); D: axial CBCT view of the left side (B23577L). The virtual planned trajectories are shown in blue, and the postoperative pterygoid screw positions are shown in white. The observed angular deviations demonstrate the accuracy of the prosthetic-guided placement protocol.

Following registration, both the planned screw models and the clinical post-implantation CBCT data were imported into 3D Slicer (version 5.8; open-source software for medical image analysis, USA) for quantitative analysis. For each pterygoid screw, angular deviation between the planned and clinical positions was measured in the screw mid-plane with respect to the sagittal and axial anatomical planes. In addition, the linear offset of the screw tip between the planned and clinical positions was quantified. All measurements were performed directly within the 3D Slicer environment (Figure 14) [12].

Table 1 summarizes the angular and linear deviations between the planned and postoperative positions of the pterygoid anchorage screws on the right (B23577R) and left (B23577L) sides.

**Table 1:** Results of the measurement of the mean deviation, expressed in millimeters and degrees, between the planned and postoperative positions of the pterygoid anchorage screws.

		B23577R	B23577L
Angle deviation (°)	Sagittal Plane	2.3°	3.7°
	Axial Plane	1.3°	1.7°
Screw tip deviation (mm)		0.74mm	1.25mm

For the right pterygoid screw (B23577R), the angular deviation was 2.3° in the sagittal plane and 1.3° in the axial plane, with a screw tip deviation of 0.74 mm.

For the left pterygoid screw (B23577L), slightly higher deviations were observed, with an angular deviation of 3.7° in the sagittal plane and 1.7° in the axial plane, and a screw tip deviation of 1.25 mm.

## Discussion

The resurgence of digitally designed custom subperiosteal implants has been enabled by substantial improvements in framework adaptation, reduced structural bulk, and enhanced accuracy provided by CAD workflows and additive manufacturing techniques. These developments have addressed several historical limitations of cast subperiosteal implants, including poor fit, excessive metal volume, and high rates of mechanical and biological complications, and recent series report encouraging survival rates and manageable complication profiles in severely atrophic jaws [17].

In the present case, during surgery, a dedicated osteotomy guide was used to contour the premaxillary ridge and shape a precise slot to accommodate the support pillars. This controlled bone preparation enabled the pillars to be seated within a predefined channel rather than resting on an irregular or convex surface. By optimizing the bone-implant contact area and reducing focal pressure on the overlying soft tissues, this strategy was intended to improve the primary mechanical integration of the subperiosteal frameworks and reduce the risk of postoperative soft-tissue recession, notably in the premaxillary region [18].

The use of the pterygomaxillary region as an anchorage site is well established in the context of pterygoid endosseous implants, where it provides dense cortical support and reduces the need for posterior grafting. Recent classifications [7] and cohort studies [19] have clarified the anatomic variations and optimal trajectories for pterygomaxillary implants. However, the anatomical complexity of this region, including the proximity of the descending palatine artery, pterygoid venous plexus, and pterygopalatine fossa, demands careful preoperative assessment and precise intraoperative execution.

Static and dynamic guided surgery have been shown to improve implant placement accuracy in atrophic jaws, including challenging pterygoid and zygomatic sites, by reducing angular and linear deviations compared with conventional freehand approaches [20]. Reported mean angular errors for guided pterygoid implants typically fall within a few degrees, with limited linear discrepancies at the entry and apex, and are considered clinically acceptable for the rehabilitative scenario [21]. In this context, the present technique extends the concept of guidance by integrating the guiding element directly into the definitive prosthetic structure, rather than relying on a separate bone-supported template or a navigation system. Once the framework is stabilized on basal bone pillars, the attached bridge provides a rigid reference for guided pterygoid osteotomy.

The choice to adopt a half-tube instead of a fully cylindrical sleeve was driven by anatomical and practical considerations. Given the extreme distal position of the pterygomaxillary access and the frequent limitation of oral opening in elderly patients with maxillary atrophy, a complete cylindrical guide would risk impeding entry, visibility, and instrument maneuverability. In addition, the apical initiation of the osteotomy is initially guided by the subperiosteal implant itself, as the implant structure incorporates a pre-planned hole intended to receive the fixation screw and thus provides inherent trajectory stabilization during the later phase of drilling. Under these conditions, partial guidance through a half-tube appeared to represent a more pragmatic compromise, facilitating introduction and orientation of the drill within a “safe corridor” while preserving access and maneuverability. Despite the absence of circumferential constraint, the deviation measured in this case remained minimal, suggesting that this simplified configuration may be sufficient to achieve the primary objective of secure and anatomically respectful pterygoid access.

The minimal deviations observed (angular deviations of 1.3° to 3.7° and linear deviations of 0.74 to 1.25 mm) demonstrate a high level of concordance between the planned and postoperative screw positions, with angular deviations remaining below 4° and linear deviations below 1.5 mm on both sides. These values are consistent with the accuracy ranges reported for guided posterior implant placement and suggest that the guided pterygoid anchorage protocol used in this case may provide reliable implant positioning in similar clinical situations. However, further clinical studies are required to validate these findings [21].

This case illustrates the feasibility of a prosthetic-guided pterygoid anchorage approach in conjunction with a modern custom subperiosteal implant for the rehabilitation of severe maxillary atrophy.

Despite these promising findings, the present work remains a single-case technical report and cannot provide high-level evidence regarding the generalizability, long-term outcomes, or comparative performance of this approach. Larger series incorporating systematic 3D accuracy assessments, complication rates, patient-reported outcomes, and clinical follow-up will be required to confirm the reproducibility of prosthetic-integrated pterygoid guidance, to benchmark its accuracy against established static and dynamic systems, and to refine indications, design parameters, and limitations for its use in different patterns of maxillary atrophy.

## Conclusions

Prosthetic-guided pterygoid drilling using an integrated half-tube guide within a definitive HUG® subperiosteal full-arch prosthesis appears feasible, accurate, and safe in the context of severe maxillary atrophy managed without the need for prior bone grafting before subperiosteal implant placement. This technique may help reduce the risks associated with free-hand pterygomaxillary access while maintaining the advantages of custom digital subperiosteal rehabilitation. Larger and longer-term clinical investigations are required to confirm these preliminary observations.

Within the limitations of a single-case report, this prosthetic-integrated guidance concept appears to be a feasible and safe option for controlling pterygoid drilling in conjunction with custom HUG® subperiosteal implants in severely atrophic maxillae. By combining a digitally planned subperiosteal framework with a semi-cylindrical guidance channel embedded in the definitive full-arch prosthesis, the technique provides a rigid reference for pterygoid osteotomy while preserving distal access and instrument maneuverability. This approach may help reduce the risks associated with freehand pterygomaxillary access. Further clinical studies with larger cohorts and standardized 3D accuracy analyses are required to confirm these preliminary observations.
